# Efficient approximation of reliabilities for single-step genomic best linear unbiased predictor models with the Algorithm for Proven and Young

**DOI:** 10.1093/jas/skab353

**Published:** 2021-12-18

**Authors:** Matias Bermann, Daniela Lourenco, Ignacy Misztal

**Affiliations:** Department of Animal and Dairy Science, University of Georgia, Athens, GA, USA

**Keywords:** accuracy approximation, BIF accuracy, genomic evaluation, prediction error variance, large-scale evaluation

## Abstract

The objectives of this study were to develop an efficient algorithm for calculating prediction error variances (**PEV**s) for genomic best linear unbiased prediction (**GBLUP**) models using the Algorithm for Proven and Young (**APY**), extend it to single-step GBLUP (**ssGBLUP**), and apply this algorithm for approximating the theoretical reliabilities for single- and multiple-trait models in ssGBLUP. The PEV with APY was calculated by block sparse inversion, efficiently exploiting the sparse structure of the inverse of the genomic relationship matrix with APY. Single-step GBLUP reliabilities were approximated by combining reliabilities with and without genomic information in terms of effective record contributions. Multi-trait reliabilities relied on single-trait results adjusted using the genetic and residual covariance matrices among traits. Tests involved two datasets provided by the American Angus Association. A small dataset (Data1) was used for comparing the approximated reliabilities with the reliabilities obtained by the inversion of the left-hand side of the mixed model equations. A large dataset (Data2) was used for evaluating the computational performance of the algorithm. Analyses with both datasets used single-trait and three-trait models. The number of animals in the pedigree ranged from 167,951 in Data1 to 10,213,401 in Data2, with 50,000 and 20,000 genotyped animals for single-trait and multiple-trait analysis, respectively, in Data1 and 335,325 in Data2. Correlations between estimated and exact reliabilities obtained by inversion ranged from 0.97 to 0.99, whereas the intercept and slope of the regression of the exact on the approximated reliabilities ranged from 0.00 to 0.04 and from 0.93 to 1.05, respectively. For the three-trait model with the largest dataset (Data2), the elapsed time for the reliability estimation was 11 min. The computational complexity of the proposed algorithm increased linearly with the number of genotyped animals and with the number of traits in the model. This algorithm can efficiently approximate the theoretical reliability of genomic estimated breeding values in ssGBLUP with APY for large numbers of genotyped animals at a low cost.

## Lay Summary

The estimated breeding value (EBV) of an animal measures its genetic merit. For calculating EBVs, pedigree and genomic information are jointly used in a procedure called single-step genomic best linear unbiased prediction (ssGBLUP). Genetic evaluations report each EBV with its reliability, which measures how accurate the breeding value estimation was. Calculating EBV with ssGBLUP for large datasets is computationally expensive; Therefore, the Algorithm for Proven and Young (APY) was developed to reduce its computational cost. However, the procedure for obtaining the reliabilities of EBV is still computationally unfeasible to apply. Thus, this study aimed to develop a new method for approximating reliabilities for ssGBLUP with APY for large datasets. We required this new method to be accurate and with fewer computational requirements than the estimation of breeding values by itself. The method that we develop consists of accumulating pedigree and genomic information in successive steps, allowing for computational efficiency. Using a dataset with more than 300,000 genotypes in a pedigree of 10,000,000 animals provided by the American Angus Association, we showed that our proposed method is accurate and computationally efficient, with a correlation of 0.98 between the approximated and target values running in less than 12 min.

## Teaser

Calculating estimated breeding values’ reliabilities in large-scale genetic evaluations is unfeasible due to heavy computations. We developed an accurate and computationally efficient method for approximating these reliabilities in a reasonable amount of time. Using our method, calculating reliabilities is no longer a bottleneck in genetic evaluations.

## Introduction

Reliabilities obtained from the inverse of the mixed model equations (**MME**s) are used for measuring the variation in the estimated breeding values (**EBV**s) from genetic evaluations; therefore, their calculation is necessary. Under the correct model specification, the reliabilities can be calculated as a function of the prediction error variances (**PEV**s), which are obtained from the diagonal of the inverse of the coefficient matrix of the MMEs (Henderson, 1984). The direct calculation of PEVs is computationally demanding because its cost increases cubically with the number of equations. Before the genomic era, several algorithms were developed for approximating PEVs (e.g., Misztal and Wiggans, 1988; Meyer, 1989; Tier et al., 1991) or their functions (e.g., Harris and Johnson, 1998; Tier and Meyer, 2004; Liu et al., 2004) by exploiting the sparse structure of the animal model (Quaas, 1976). However, with the use of dense panels of single-nucleotide polymorphism (**SNP**) for genetic evaluations, such a structure was lost, and the existing algorithms were not useful.

For situations where all the animals are genotyped, the existing methods for estimating reliabilities rely on the equivalence between breeding value (**GBLUP**) and marker effect models (SNP-BLUP; Liu et al., 2014). The reason for this is that for the latter, the dimension of the coefficient matrix of the MMEs remains constant with the number of genotyped animals. However, this advantage is lost when a residual polygenic effect is added to the model (Liu et al., 2014). Therefore, different strategies such as reducing the number of SNP, extracting a subset of reference animals (Sargolzaei et al., 2014), or using Monte Carlo sampling (Ben Zaabza et al., 2020) were developed for reducing the computational burden of approximating genomic reliabilities. It is worth noting that all of these methods have a computational cost that increases cubically with the number of traits and SNPs, and quadratically with the number of genotyped animals.

When not all the animals are genotyped, methods such as single-step GBLUP (**ssGBLUP**; Aguilar et al., 2010), single-step SNP-BLUP (Liu et al., 2014), or single-step Bayesian Regression (Fernando et al., 2014) are required for estimating breeding values for all genotyped and non-genotyped animals. In such a case, direct inversion of a block of the coefficient matrix of the MMEs (Misztal et al., 2013), propagation of genomic information via reverse reliability calculations (Liu et al., 2017; Edel et al., 2019), or Monte Carlo sampling (Fernando et al., 2016) was proposed for calculating PEV. On the one hand, the first two types of methods have an equal computational complexity than the procedures for estimating reliabilities for a GBLUP model, because the reliability for a GBLUP model is estimated as an intermediate step for single-step estimation methods. On the other hand, convergence and elapsed time per sample are the major drawbacks of Monte Carlo sampling methods (Hickey et al., 2009; Fernando et al., 2016). Therefore, their application to large datasets is very time-consuming.

Although large-scale genomic evaluations are feasible with the Algorithm for Proven and Young (**APY**; Misztal, 2016), which relies on the sparse representation of the inverse of the genomic relationship matrix, no specific methods exist for calculating the reliability when using APY. Therefore, the objectives of this study were to: 1) develop an efficient method for calculating reliabilities for APY-GBLUP models and extend it to APY-ssGBLUP and 2) apply the algorithm for estimating reliabilities in ssGBLUP single- and multiple-trait models.

## Materials and Methods

We present the algorithm in two sections for easy understanding: a new GBLUP-based approach to combine genomic information and records contributions, and the propagation to ssGBLUP using existing techniques.

### Single-trait APY-GBLUP

Let an APY-GBLUP model be: 


$$\begin{array}{l} y=Xb+Wu+e \end{array}$$

$$\begin{array}{l} E\left[y\right]=Xb \end{array}$$

$$\begin{array}{l} Var\left[\begin{array}{l} u\\ e \end{array}\right]=\left[\begin{array}{cc} G_{APY}\sigma_{u}^{2} & 0\\ 0 & I\sigma_{e}^{2} \end{array}\right] \end{array}$$ (1)

where y is the vector of phenotypes, b is the vector of fixed effects, e is the vector of error terms, X and W are incidence matrices, and $\sigma_{u}^{2}$ and $\sigma_{e}^{2}$ are the genetic and residual variances, respectively. The structure of $G_{APY}$ and its inverse, as defined in Misztal (2016), is:


$$\begin{array}{ll} G_{APY}= & \left[\begin{array}{cc} I & 0\\ P_{nc} & I \end{array}\right]\left[\begin{array}{cc} G_{cc} & 0\\ 0 & M_{nn} \end{array}\right]\left[\begin{array}{cc} I & P_{cn}\\ 0 & I \end{array}\right]\\ & =\left[\begin{array}{cc} G_{cc} & G_{cn}\\ G_{nc} & M_{nn}+G_{nc}G_{cc}^{-1}G_{cn} \end{array}\right] \end{array}$$

$$\begin{array}{l} G_{APY}^{-1}=\left[\begin{array}{cc} I & -P_{cn}\\ 0 & I \end{array}\right]\left[\begin{array}{cc} G_{cc}^{-1} & 0\\ 0 & M_{nn}^{-1} \end{array}\right]\left[\begin{array}{cc} I & 0\\ -P_{nc} & I \end{array}\right]\\ =\left[\begin{array}{cc} G_{cc}^{-1}+P_{cn}M_{nn}^{-1}P_{nc} & -P_{cn}M_{nn}^{-1}\\ -M_{nn}^{-1}P_{nc} & M_{nn}^{-1} \end{array}\right]=\left[\begin{array}{cc} G^{cc} & G^{cn}\\ G^{nc} & M_{nn}^{-1} \end{array}\right] \end{array}$$ (2)

where the subscripts *c* and *n* represent the core and noncore animals, respectively; $P_{nc}=G_{nc}G_{cc}^{-1}$; $P_{cn}=G_{cc}^{-1}G_{cn}$; and $M_{nn}=diag\left(G_{nn}-G_{nc}G_{cc}^{-1}G_{cn}\right)$ is a diagonal matrix. Letting $\alpha =(\sigma_{e}^{2})/(\sigma_{u}^{2})$, the MMEs for the model (1) are:


$$\begin{array}{l} \left[\begin{array}{cc} X{}^{\prime }\;X & X{}^{\prime }\;W\\ W{}^{\prime }\;X & W{}^{\prime }\;W+G_{APY}^{-1}\alpha \end{array}\right]\left[\begin{array}{l} b\;\\ u\; \end{array}\right]=\left[\begin{array}{c} X{}^{\prime }\;y\\ W{}^{\prime }\;y \end{array}\right] \end{array}$$ (3)

Then, the PEVs are obtained from:


$$\begin{array}{l} diag{\left(D+G_{APY}^{-1}\alpha \right)}^{-1} \end{array}$$ (4)

where D is a diagonal matrix such that $D\approx W^{\prime }\left(I-X{\left(X^{\prime }X\right)}^{-1}X^{\prime }\right)W$ (VanRaden and Freeman, 1985; Misztal and Wiggans, 1988).

Here, we assume that $G_{APY}^{-1}$ is created following the block implementation in Masuda et al. (2016) and stored in disk. For obtaining equation (4), we implemented an algorithm for calculating a block sparse inverse, following formula (8) from Henderson and Searle (1981). Then, the steps for calculating the PEV are (A1):

1. Read and store $G_{APY}^{-1}$ in memory;
2. Approximate D and overwrite $G_{APY}^{-1}$ as$G_{APY}^{-1}=D+G_{APY}^{-1}\alpha$. Note that this implies updating the diagonal elements of $G^{cc}$ and the diagonal matrix $M_{nn}^{-1}$;
3. Calculate $G^{cn^{*}}=G^{cn}M_{nn}$;
4. Overwrite $G^{cc}$as $G^{cc}=G^{cc}-G^{cn}(G^{cn^{*}}){\;}^{\prime }\;$;
5. Invert $G^{cc}$;
6. Overwrite $G^{cn}$ as $G^{cn}=-G^{cc}G^{cn^{*}}$;
7. Overwrite $M_{nn}^{-1}$ as $M_{nn_{i}}^{-1}=M_{nn_{i}}-{\left(G_{.i}^{cn^{*}}\right)}^{{\;}^{\prime }\;}G_{.i}^{cn}$, where the subscript *i* refers to the *ith* element of $M_{nn}^{-1}$ and $M_{nn}$, or the *ith* row of $G^{cn^{*}}$ and $G^{cn}$;
8. Obtain PEVs from $diag\left(G^{cc}\right)$ and $M_{nn}^{-1}$ for core and noncore animals, respectively.

### Algorithm for Proven and Young-ssGBLUP

The method presented for single-trait APY-GBLUP can be used for approximating the reliabilities for a single-trait APY-ssGBLUP by using different procedures based on effective records contributions (**ERC**s) such as in Liu et al. (2017) or Edel et al. (2019), among others. A general outline of the method is (A2):

1. Approximate pedigree reliabilities and obtain ERCs.
2. For genotyped animals, solve equation (4) in A1 using ERCs instead of D.
3. Approximate pedigree reliabilities for genotyped animals without taking into account the information provided by non-genotyped animals by removing their contributions. This will be referred to as approximating reliabilities of $A_{22}$, where $A_{22}$ stands for the numerator relationship matrix for genotyped animals.
4. Obtain final reliabilities for genotyped animals using formulas (18 to 27) from Liu et al. (2017).
5. Back-solve the reliabilities for genotyped animals to get ERCs. A detailed procedure for back-solving the reliabilities to obtain ERCs is explained in the Supplementary Appendix. Then, calculate reliabilities for non-genotyped animals by applying those ERCs as weights in a method for obtaining pedigree reliabilities.

### Extension to multiple-trait models

For extending both single-trait APY-GBLUP and APY-ssGBLUP to multi-trait models, single-trait reliabilities were adjusted using the genetic and residual covariance matrices among traits following the method of Strabel et al. (2001). For each animal, this method requires a diagonal matrix with effective observations per trait (O) obtained from the single-trait reliabilities, the matrices of genetic $\left(G_{0}\right)$ and residual (**R**_0_) covariances among traits, and the number of progenies for each trait. Then, for each animal, its adjusted reliability for the *jth* trait is equal to:


$$\begin{array}{l} rel_{j}=1-\frac{W_{jj}}{G_{0_{jj}}} \end{array}$$ (5)

Where,


$$\begin{array}{l} W={\left({\left(O^{0.5}R_{0}O^{0.5}\right)}^{-1}+G_{0}^{-1}+\underset{j}{\overset{n_{traits}}{\sum }}\;\frac{1}{3}m_{j}G_{0}^{-1}-\frac{2}{3}m_{j}G_{0}^{-1}{\left(\frac{4}{3}G_{0}^{-1}+{\left(QR_{0}Q\right)}^{-1}\right)}^{-1}\frac{2}{3}G_{0}^{-1}\right)}^{-1} \end{array}$$ (6)

and $n_{traits}$ is the number of traits, $m_{j}$ is the number of progenies for the *jth* trait, and is Q a diagonal matrix with $Q_{jj}=1$if the *jth* trait is non-missing and zero otherwise. It is worth noticing that the dimension of all the matrices in equation (6) is equal to the number of traits. Therefore, calculating equation (6) is not computationally demanding. For more details, we refer the reader to Strabel et al. (2001).

### Implementation

For calculating the reliabilities of APY-GBLUP, only the nonzero elements of $G_{APY}^{-1}$ were stored in disk, as suggested by Masuda et al. (2016). The approximation of **D** requires reading the data file only twice. Although Harris and Johnson (1998) suggested to adjust records only for the major fixed effect, we approximated **D** by accounting for all the cross-classified effects, which can be more robust. The inversion of $M_{nn}^{-1}$ is straightforward since it is a diagonal matrix. The matrix multiplications were performed using the dgemm subroutine from the Intel Math Kernel Library (**MKL**; Intel corporation), whereas the inversion of $G^{cc}$ was performed using dpotrf and dpotri from the same library. All the MKL subroutines and loops were parallelized using OpenMP (http://www.openmp.org). The most time-consuming steps from Algorithm A1 are 4 to 6. Letting $n_{c}$ be equal to the number of core animals and $n_{n}$ be the number of noncore animals, the computational costs of steps 4 to 6 without any optimized algorithm are $O\left(n_{c}^{2}n_{n}\right)$, $O\left(n_{c}^{3}\right)$, and$O\left(n_{c}^{2}n_{n}\right)$, respectively. The notation O(f(n)) denotes that a function is upper bounded by f(n) when n→∞ (Knuth, 1976). Thus, for example, an algorithm with asymptotic behavior O(n) is preferable to another whose limiting behavior is $O\left(n^{2}\right)$. Although step 5 requires matrix inverse, the number of core animals in APY is hardly ever over 25k (Pocrnic et al., 2016).

For approximating the reliabilities under APY-ssGBLUP, the animals should be ordered such that the parents precede their progeny for obtaining ERCs (Harris and Johnson, 1998). This was implemented recursively by first classifying the animals in nonoverlapping generations using a parallelized subroutine (with OpenMP) and then by ordering using a recursive Quicksort (Sedgewick, 1990; pp. 118). The default pedigree reliabilities were approximated by the method of Harris and Johnson (1998). In this case, ERCs are obtained as a subproduct of the reliability estimation and calculated following Liu et al. (2018) but considering the own record’s contributions for males, which the authors did not consider since they presented their formulae for dairy cattle. However, the user can provide external pedigree reliabilities that could be back-solved using a root-finding technique to obtain ERCs using the method of Liu et al. (2018) (see Supplementary Appendix). The root-finding technique that we chose was Steffensen’s method (Johnson and Scholz, 1968) because it does not require derivatives, has good convergence properties, and its implementation is straightforward. For calculating genomic reliabilities, Algorithm A1 was used as described in the previous paragraph. Finally, the propagation to the non-genotyped animals was implemented by back-solving the reliabilities for genotyped animals using Steffensen’s method. The implementation of the adjustment for multiple traits used the native functions from Fortran because the matrix products and inversions are of dimension equal to the number of traits.

### Data

The two datasets used in this study to test the approximated reliabilities of genomic EBV from APY-ssGBLUP were provided by the American Angus Association (St. Joseph, MO). A small dataset (Data1) was used for comparing the approximated reliabilities with the reliabilities obtained by the inversion of the left-hand side of the MMEs. A large dataset (Data2) was used for evaluating the computational performance of the algorithm. Data1 for single-trait analysis (Data1_st) consisted of 50,000 animals that had genotypes for 39,759 SNPs after quality control. From the genotyped animals, 10,523 were randomly selected as core based on the number of core animals proposed by Pocrnic et al. (2016) for different livestock species. This number of core animals was proposed based on the number of eigenvalues explaining 98% of the variance in the spectrum of the genomic relationship matrix. Consequently, the number of noncore animals was 39,477. The number of core animals was the same across all the datasets and analyses, as well as the number of SNPs. In Data1_st, the pedigree consisted of 167,951 animals, of which 76,758 had records for postweaning gain (**PWG**). In Data1 used for multi-trait analysis (Data1_mt), 78,641 animals had phenotypes for at least one of the three traits: PWG, birth weight (**BW**), or weaning weight (**WW**), and the number of animals in the pedigree was 172,089. Because of limitation in the computation of the inverse of the left-hand side of the MMEs for multi-trait models with many genotypes, the total number of genotyped animals in Data1_mt had to be reduced to 20,000, of which 9,477 were noncore.

The second dataset (Data2) had 335,325 genotyped animals, and the number of noncore animals was equal to 324,802. In single-trait analysis (Data2_st), 4,218,407 individuals had records for PWG, and the total number of animals in the pedigree was 10,213,401. The multi-trait analysis with Data2 (Data2_mt) used the same number of animals in the pedigree as in Data2_st, but 8,681,659 animals had phenotypes for at least one of the three traits. A summary of all datasets can be found in Table 1.

**Table 1.** Traits, number of animals in the pedigree, number of animals with records, and number of genotyped animals for each dataset

		Data1^1^		Data2^1^	
		Data1_st	Data1_mt	Data2_st	Data2_mt
Trait(s)^2^		PWG	BW—WW—PWG	PWG	BW—WW—PWG
Animals in the pedigree		167,951	172,089	10,213,401	10,213,401
Animals with records		76,758	78,641	4,218,407	8,681,659
Genotyped animals	Core	10,523	10,523	10,523	10,523
	Noncore	39,477	9,477	324,802	324,802

^1^Data1_st , Data1 for single-trait analysis; Data1_mt, Data1 used for multi-trait analysis; Data2_st , Data2 for single-trait analysis; Data2_mt, Data2 used for multi-trait analysis.

^2^The traits are birth weight (BW), weaning weight (WW), and postweaning gain (PWG).

### Software, computational resources, and benchmark tests

PreGSf90 (Misztal et al., 2014) was used to calculate and store $G_{APY}^{-1}$. The exact reliabilities for Data1_st and Data1_mt were obtained by sparse inversion of the Cholesky factor of the coefficient matrix using BLUPF90. Those reliabilities were used as benchmark to compare the approximation algorithm presented here. Comparisons included the correlation between exact and approximated reliabilities, the intercept and slope of the regression of exact on the approximated reliabilities, and the mean absolute change (i.e., the absolute difference between exact and approximated reliability). Because the novelty of this approximated reliability is to use $G_{APY}^{-1}$ instead of $G^{-1}$ in the computation of $diag{\left(D+G_{APY}^{-1}\alpha \right)}^{-1}$, we also investigated reliabilities when using $G^{-1}$.

The methods presented in the previous sections were programmed in Fortran 95 and compiled with the Intel Fortran Compiler version 15.0.3 with options -fpp and -O3. All the computations were performed on a Dell PowerEdge R740XD server with 1.5 TB of memory, 45 TB of disk, and two Intel Xeon Gold 6258R processors with 56 threads each; however, the number of threads for all the computations was limited to 32.

## Results and Discussion

**Figure 1.**
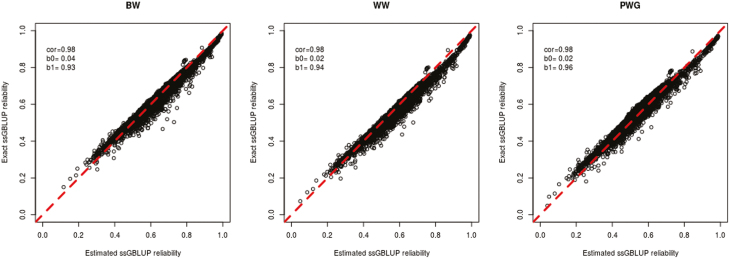
Scatter plots comparing reliability obtained from the inverse of the mixed model equation against estimated reliability for the genotyped animals in Data1_mt. Abbreviations: BW, birth weight; PWG, postweaning gain; ssGBLUP, single-step genomic best linear unbiased prediction; WW, weaning weight.

Table 2 presents statistics for the approximated reliabilities for Data1_st and Data1_mt. For both genotyped and non-genotyped animals, correlations were above 0.97. Also, for all the scenarios, the mean absolute change was smaller than 0.03. The slope of the regression of the true on the approximated reliabilities for the genotyped animals ranged from 0.93 to 0.96, whereas the intercept ranged from 0.02 to 0.04. For non-genotyped animals, the slope ranged from 0.97 to 1.05, whereas the intercept ranged from 0 to 0.01. However, the mean square errors were significantly higher for the non-genotyped animals (results not shown). Scatter plots for the comparison between true and estimated reliabilities for genotyped animals for the Data1_mt are shown in Figure 1. It can be noticed that there is a minor overestimation of the reliabilities for BW and WW. However, the fit for the three models is appropriate and, consequently, the method is accurate. This overestimation was due to a slight overestimation of the pedigree reliabilities from the first step in A2.

**Table 2.** Correlation, intercept, slope, and mean absolute change (MAC) between the exact and estimated reliabilities for Data1_st and Data1_mt

Dataset	Trait^1^	Group	Correlation	Intercept	Slope	MAC
Data1_st^2^	PWG	Genotyped	0.98	0.02	0.94	0.01
		Non-genotyped	0.97	0.01	1.05	0.03
Data1_mt^2^	BW	Genotyped	0.98	0.04	0.93	0.01
		Non-genotyped	0.98	0.00	0.98	0.02
	WW	Genotyped	0.98	0.02	0.94	0.01
		Non-genotyped	0.99	0.00	0.97	0.02
	PWG	Genotyped	0.98	0.02	0.96	0.01
		Non-genotyped	0.99	0.00	1.01	0.01

^1^BW, birth weight; PWG, postweaning gain; WW, weaning weight.

^2^Data1_st , Data1 for single-trait analysis; Data1_mt, Data1 used for multi-trait analysis.

The comparison between reliabilities obtained from inversion of the MME with $G^{-1}$ and $G_{APY}^{-1}$ is presented in Table 3. All correlations were greater than 0.98 for both Data1_st and Data1_mt for genotyped and non-genotyped animals. In all cases, the reliabilities for non-genotyped animals were almost identical. However, for genotyped animals, the slope and the intercept of the regression of true reliability with $G^{-1}$ on the true reliability with $G_{APY}^{-1}$ for Data1_st were 0.92 and 0.06, respectively. For the multiple-trait model, that is for Data1_mt, the slopes were greater than the unity, ranging from 1.05 to 1.10; the intercepts were lower than zero, ranging from −0.03 to −0.07. According to these results, it is not possible to state that the reliability is under or overestimated with APY when compared with $G^{-1}$.

**Table 3.** Correlation, intercept, slope, and mean absolute change (MAC) between the reliabilities obtained by inversion with $G^{-1}$ and $G_{APY}^{-1}$ for Data1_st and Data1_mt

Dataset	Trait^1^	Group	Correlation	Intercept	Slope	MAC
Data1_st^2^	PWG	Genotyped	0.99	0.06	0.92	0.01
		Non-genotyped	0.99	0.00	1.00	1.0 × 10^-3^
Data1_mt^2^	BW	Genotyped	0.98	−0.03	1.05	0.01
		Non-genotyped	0.98	0.00	0.99	0.02
	WW	Genotyped	0.98	−0.03	1.07	0.01
		Non-genotyped	0.98	0.00	0.98	0.02
	PWG	Genotyped	0.98	−0.07	1.10	0.01
		Non-genotyped	0.99	0.00	0.99	0.02

^1^BW, birth weight; PWG, postweaning gain; WW, weaning weight.

^2^Data1_st , Data1 for single-trait analysis; Data1_mt, Data1 used for multi-trait analysis.

Table 4 presents the elapsed wall clock time for each step of Algorithm A2 for each dataset. The total time for the largest dataset in this study (i.e., Data2_mt) was 11 min. It can be observed that the computing time for A1 (i.e., to obtain $diag{\left(D+G_{APY}^{-1}\alpha \right)}^{-1}$) increases proportionally with the number of genotyped animals. As an example, for the single-trait models, the number of genotyped animals increased seven times when moving from Data1_st to Data2_st, whereas the elapsed time for A1 also increased by seven times. For the multiple-trait models, the increase in wall clock time for A1 was less than proportionally. While the amount of genotyped animals increased 16-fold when moving from Data1_mt to Data2_mt, the elapsed time only increased 14-fold. On the other hand, an increase in the number of traits changed the elapsed time a little more than proportionally. For instance, when comparing Data2_st against Data2_mt, the number of traits increased by three but the total elapsed time increased by 3.3 times. The reason for this is that for multiple-trait models, not only the single-trait reliabilities but also the adjustment for multiple traits is required (Strabel et al., 2001). When subtracting the latter step from the algorithm, the total elapsed time increased less than proportionally with the number of traits. It is worth noticing that this comparison cannot be done with Data1_st and Data2_st because of the different number of genotyped animals. Finally, it can be observed that the elapsed time for the pedigree reliability estimation increased less than proportionally with the number of animals in the pedigree.

**Table 4.** Wall clock time in minutes of each step for estimating reliabilities for each dataset^1^

	Single trait		Multiple trait	
	Data1 (7.5 GB)	Data2 (55 GB)	Data1 (2.7 GB)	Data2 (55 GB)
Sorting pedigree	0.003	0.23	0.003	0.23
Approximation of pedigree reliabilities	0.009	0.46	0.031	1.37
Calculation of GBLUP^2^ reliabilities	0.28	1.85	0.36	4.92
Approximation of reliabilities of $A_{22}$^3^	0.008	0.44	0.025	1.32
Propagation to non-genotyped animals	0.001	0.05	0.002	0.16
Multiple-trait adjustment	—	—	0.03	3.23
Total time	0.31	3.32	0.42	11.11

^1^The memory requirements in gigabytes (GB) are inside parenthesis.

^2^GBLUP, genomic best linear unbiased prediction.

^3^$A_{22}$ refers to the numerator relationship matrix for genotyped animals.

As can be noticed, the method developed in the present study is much faster than the current methods for approximating reliabilities. For example, Erbe et al. (2018) reported that the calculation of genomic reliabilities for 78,000 genotyped animals took 35 min, whereas for 222,619 genotyped animals, Ben Zaabza et al. (2020) and Ben Zaabza et al. (2021) reported a minimum of 140 and 36 min, respectively. Besides the employed hardware, algorithmic differences explain why our method is much faster than the cited references. The main contrast between these and our method is the way of calculating the genomic reliabilities. The computational complexity of those methods arises from making the MME, inverting them, and obtaining the individual reliabilities from the reliabilities of the SNP. The complexity of the first and third tasks increases quadratically with the number of markers and linearly with the number of genotyped animals, whereas the complexity of the second step increases cubically, at least, with the number of markers. If a residual polygenic effect is included in the model, then the complexity of the inversion of the system increases more than cubically with the number of markers. The algorithm from the present study does not require constructing the MME but approximating weights to be added to the diagonal of the inverse of the genomic relationship matrix. These weights are an approximation of the absorption of the equations related to fixed effects (Mohammad et al., 1985), and its approximation is not costly (VanRaden and Freeman, 1985). Then, the most expensive steps from the sparse inversion in A1 are two matrix multiplications whose computational complexity increases quadratically with the number of core animals and linearly with the number of noncore animals and a matrix inversion that increases always cubically with the number of core animals. From this, it can be observed that the advantage in elapsed time of our algorithm comes from the fact that the number of core animals (~10k to 20k in cattle) is much less than the number of markers (~30k to 80k) for routine genetic evaluations (Misztal et al., 2020) and that the matrix multiplication takes more advantage of the parallel computations than the matrix inversion.

Since the reliability estimation is done after estimating breeding values, $G_{APY}^{-1}$ can be stored in disk from the latter and be reused for the former. This is the reason that we did not consider the calculation of $G_{APY}^{-1}$ as a part of the algorithm in Table 3. Nonetheless, the wall clock time for calculating $G_{APY}^{-1}$ for Data1_st and Data1_mt was less than a minute, whereas for Data2_st and Data2_mt was 12 min. It is worth noticing that the memory requirements for approximating the reliabilities for our method are less than or equal to the memory requirements for estimating breeding values. Furthermore, with multiple traits, it is possible to read and deallocate $G_{APY}^{-1}$ (i.e., $D+G_{APY}^{-1}\alpha$) for each single-trait reliability estimation. Therefore, the memory requirements do not increase by increasing the number of traits. As presented in Table 4, the memory requirements for estimating the reliabilities were 7.5 and 3 GB for Data1_st and Data1_mt, respectively, and 55 GB for Data2_st and Data2_mt. Such values do not represent limitations for most of the servers where routine genetic evaluations are run.

## Conclusion

An efficient method for calculating exact genomic reliabilities with APY $G^{-1}$ was developed. This method is the basis of a larger procedure for approximating reliabilities for single- and multiple-trait ssGBLUP models. The algorithm proposed in this study was both accurate and computationally efficient. Since the memory requirements and computing time of the proposed method are less than or equal to the ones required for estimating breeding values, it can be applied for routine genetic evaluations. Therefore, the approximation of reliabilities for large ssGBLUP models is no longer a bottleneck in genetic evaluations.

## Supplementary Material

skab353_suppl_Supplementary_Materials

## Data Availability

The data belong to American Angus Association (Saint Joseph, IL). Therefore, they cannot be shared.

## Abbreviations

APY: Algorithm for Proven and Young
BW: birth weight
EBV: estimated breeding value
ERC: effective records contribution
GBLUP: genomic best linear unbiased predictor
MME: mixed model equation
PEV: prediction error variance
PWG: postweaning gain
SNP: single-nucleotide polymorphism
SNP-BLUP: single-nucleotide polymorphism best linear unbiased predictor
ssGBLUP: single-step GBLUP;
WW: weaning weight
