# Supplementary material for: Efficient approximation of reliabilities for single-step genomic best linear unbiased predictor models with the Algorithm for Proven and Young
Source: J Anim Sci. 2021 Dec 18;100(1):skab353. doi: 10.1093/jas/skab353 (PMC8827023; doi:10.1093/jas/skab353)
Supplement: skab353_suppl_Supplementary_Materials [file skab353_suppl_Supplementary_Materials.docx]

**Appendix**

Here, we explain how to obtain effective records contributions (ERC) as in Liu et al. (2018) from external reliabilities provided by the user. In Liu et al. (2018), the ERC are obtained based on each animal’s reliability based on its own records $\left( R\left( o \right) \right)$. Thus, it suffices to calculate $R\left( o \right)$ for each animal in the pedigree.

As defined by Harris & Johnson (1998), the *i^th^* animal’s reliability $\left( {R\left( pa+o+p \right)}_{i} \right)$ is equal to:

| ${R\left( pa+o+p \right)}_{i}=\frac{{R\left( o+p \right)}_{i}+{R\left( \mathrm{pa} \right)}_{i}-2 {R\left( \mathrm{pa} \right)}_{i} {R\left( o+p \right)}_{i}}{1-{R\left( \mathrm{pa} \right)}_{i} {R\left( o+p \right)}_{i}}$ | (1.1) |
| --- | --- |

where ${R\left( o+p \right)}_{i}$ is the reliability combining progeny and own records contributions, and ${R\left( \mathrm{pa} \right)}_{i}$ is the reliability of parent average. Also:

| ${R\left( \mathrm{pa} \right)}_{i}=0.25 \left( R_{\mathrm{sire}}\left( \mathrm{pt} \right)+R_{\mathrm{dam}}\left( \mathrm{pt} \right) \right)$ | (1.2) |
| --- | --- |

where

| $R_{sire(dam)}\left( \mathrm{pt} \right)=\frac{{R\left( pa+o+p \right)}_{sire(dam)}-0.25 {R\left( o+p \right)}_{i}}{1-0.5 {R\left( o+p \right)}_{i} + 0.25 {R\left( o+p \right)}_{i} {R\left( pa+o+p \right)}_{sire(dam)}}$ | (1.3) |
| --- | --- |

By observing (1.2) and (1.3), it can be noted that the equation (1.1) is a function of ${R\left( o+p \right)}_{i}$. Thus, Steffensen’s method (Johnson & Scholz, 1968) or any root-finding method can be used to solve:

| $0=\frac{{R\left( o+p \right)}_{i}+{R\left( \mathrm{pa} \right)}_{i}-2 {R\left( \mathrm{pa} \right)}_{i} {R\left( o+p \right)}_{i}}{1-{R\left( \mathrm{pa} \right)}_{i} {R\left( o+p \right)}_{i}}-{R\left( pa+o+p \right)}_{i}$ | (1.4) |
| --- | --- |

for ${R\left( o+p \right)}_{i}$. Then, ${R\left( o \right)}_{i}$ is obtained as

| ${R\left( o \right)}_{i}=\frac{{R\left( o+p \right)}_{i}-{R\left( p \right)}_{i}}{1+{R\left( p \right)}_{i} {R\left( o+p \right)}_{i}-2 {R\left( p \right)}_{i}}$ | (1.5) |
| --- | --- |

where ${R\left( p \right)}_{i}$ is the reliability based on progeny, which is calculated with Equations (4) and (5) from Harris & Johnson (1998).

The overall procedure is as follows:

1. Calculate ${R\left( p \right)}_{i}$ following Step 2 and Equations (4-5) from Harris & Johnson (1998). This step only requires the heritability and number of progeny with records are required.
2. From the oldest to the youngest animal in the pedigree:
   1. If parents are known, retrieve their reliabilities from memory.
   2. Obtain ${R\left( o+p \right)}_{i}$ from (1.4) using a root-finding method.
   3. Obtain ${R\left( o \right)}_{i}$ from (1.5).
3. Calculate ERC following the method of Liu et al. (2018).
